# Attenuation of CCl_4_-Induced Oxidative Stress and Hepatonephrotoxicity by Saudi Sidr Honey in Rats

**DOI:** 10.1155/2013/569037

**Published:** 2013-02-24

**Authors:** Mohammed Al-Yahya, Ramzi Mothana, Mansour Al-Said, Mohammed Al-Dosari, Nawal Al-Musayeib, Mohammed Al-Sohaibani, Mohammad Khalid Parvez, Syed Rafatullah

**Affiliations:** ^1^Department of Pharmacognosy, College of Pharmacy, King Saud University, P.O. Box 2457, Riyadh 11451, Saudi Arabia; ^2^Medicinal, Aromatic and Poisonous Plants Research Center (MAPPRC), College of Pharmacy, King Saud University, P.O. Box 2457, Riyadh 11451, Saudi Arabia; ^3^Department of Pathology (32), King Khalid University Hospital, King Saud University, P.O. Box 2925, Riyadh 11461, Saudi Arabia

## Abstract

The present study was undertaken to investigate the possible protective effect of Saudi Sidr honey (SSH) on carbon tetrachloride (CCl_4_) induced oxidative stress and liver and kidney damage in rat. Moreover, the antioxidant activity and the phenolic and flavonoidal contents were determined. The hepatorenal protective activity of the SSH was determined by assessing biochemical, hematological, and histological parameters. Serum transaminases, ALP, GGT, creatinine, bilirubin urea, uric acid, and MDA level in liver and kidney tissues were significantly elevated, and the antioxidant status of nonprotein sulfhydryls, albumin, and total protein levels in liver and kidney were declined significantly in CCl_4_ alone treated animals. Pretreatment with SSH and silymarin prior to the administration of CCl_4_ significantly prevented the increase of the serum levels of enzyme markers and reduced oxidative stress. SSH also exhibited a significant lipid-lowering effect and caused an HDL-C enhanced level in serum. The histopathological evaluation of the liver and kidney also revealed that honey protected incidence of both liver and kidney lesions. Moreover, SSH showed a strong antioxidant activity in DPPH and **β**-carotene-linoleic acid assays. SSH was found to contain phenolic compounds. Additionally, the SSH supplementation restored the hepatocytes viability against 2′,7′-dichlorofluorescein (DCF) toxicity in *ex vivo* test.

## 1. Introduction

Aasal is the Arabic name for honey. It is a naturally sweet and flavorful product produced by honeybees, *Apis mellifera*, from the nectar of blossoms or from the exudates of trees and plants giving the nectar honey or honeydews [1]. Honey is a natural, unprocessed, and easily digested food in the diet [2]. Honey has been used in almost all cultures and traditions since ancient times as a food and medicinal product. It is considered to be used for the treatment of burns, faster wound healing [3], gastrointestinal disorders, asthma, skin, and eye diseases, besides being a natural food preservative and sweetening agent [4, 5]. Hippocrates, the father of medicine, describes the nutritional and medicinal importance of honey [6].

Numerous nutritional [7] and biological effects [8] are attributed to honey, which include antibacterial [9], antioxidant [10], antiviral [11], antiparasitic [12], anti-inflammatory [13], anticancer [14], and immunosuppressive [15] activities. Apart from various therapeutic uses, honey is used in many folkloric traditions, for instance it is given to new born babies to clean their digestive system. Honey is considered to be the first in the line to treat jaundice in traditional medicine of different countries. In the present investigation, an attempt has been made to validate its use in both liver and kidney disorders.

## 2. Materials and Methods 

Pure honey (Saudi Sidr variety) was purchased from an exclusive honey shop in Riyadh, Saudi Arabia.

### 2.1. Acute Toxicity Test

The acute toxicity test was performed on the mice using the oral route. SSH was dissolved in distilled water and administered at various doses, ranging from (500–5000 mg/kg), to different groups of mice. The animals were observed continuously for 1 h and then at half-hourly intervals for 4 h on the first day for clinical signs and symptoms of toxicity and further up to 72 h followed by 14 days for any mortality.

### 2.2. Animals and Study Design

Wistar albino rats (180–200 g) were obtained from the Experimental Animal Care Center of the College of Pharmacy, King Saud University, Riyadh. Animals were maintained on standard chow diet and housed in polycarbonate cages in a room free from any source of chemical contamination, artificially illuminated (12 h dark/light cycle) and thermally controlled (25 ± 2°C) at the animal facility. All animals received humane care in compliance with the guidelines of the Ethics Committee of the Experimental Animal Care Society, College of Pharmacy, King Saud University, Riyadh, Saudi Arabia.

After a one-week acclimatization period, animals were randomly allocated into 5 groups and treated as follows: group (1), untreated control; group (2, 3, 4, and 5) received 0.25 mL of CCl_4_ in liquid paraffin (1 : 1) 1.25 mL/kg body weight intraperitoneally (IP). Group 2 was administered only CCl_4_. Groups 3 and 4 received SSH 0.5 and 1.0 g/kg/day orally for 6 weeks. Rats in group 5 were treated with 10 mg/kg orally with silymarin for similar days. The blood was collected by cardiac puncture after 24 h following the administration of CCl_4_, allowed to clot and the serum was separated. After collecting the blood, the animals were sacrificed using ether anaesthesia. The liver and kidneys were dissected out and used for biochemical estimations and histological assessment.

#### 2.2.1. Estimation of Marker Enzymes and Bilirubin

Serum glutamate oxaloacetate transaminase (SGOT), serum glutamate pyruvate transaminase (SGPT) [16], alkaline phosphatase (ALP) [17], gamma-glutamyl transferase (GGT) [18], hemoglobin, and bilirubin [19] were determined using Reflotron Plus Analyzer and Roche kits (Roche Diagnostics GmbH, Mannheim, Germany).

#### 2.2.2. Estimation of Lipid Profile

Total cholesterol [20], triglycerides [21], high-density lipoproteins (HDLC) [22], and glucose levels were estimated in serum using Roche diagnostic kits (Roche Diagnostics GmbH, Mannheim, Germany).

#### 2.2.3. Determination of Malondialdehyde (MDA)

The method reported by Utley et al. [23] was followed. In brief, the liver and kidney tissues were removed, and each tissue was homogenized in 0.15 M KCl (at 4°C; Potter-Elvehjem type C homogenizer) to give a 10% w/v homogenate. The absorbance of the solution was then read at 532 nm. The content of malondialdehyde (nmol/g wet tissue) was then calculated, by reference to a standard curve of malondialdehyde solution.

#### 2.2.4. Estimation of Nonprotein Sulfhydryls (NP-SH)

Hepatic nonprotein sulfhydryls were measured according to the method of Sedlak and Lindsay [24]. The liver and kidney were homogenized in ice-cold 0.02 mmol/L ethylenediaminetetraacetic acid (EDTA). The absorbance was measured within 5 min of addition of 5,5′-dithio-bis(2-nitrobenzoic acid) (DTNB) at 412 nm against a reagent blank.

#### 2.2.5. Determination of Total Protein (TP)

Total protein was estimated by the kit method, supplied by Crescent Diagnostics, Jeddah, Saudi Arabia. The absorbance of this complex at 546 nm is proportional to the protein concentration. The serum total protein was calculated using the equation:

(1) Serum  total  protein  =ABSsample    ABSstandard×concentration  of  standard.

#### 2.2.6. Histopathological Evaluation

The liver and kidney tissue samples were fixed in neutral buffered formalin for 24 h. Sections of the liver tissue were histopathologically examined. These sections were stained with haematoxylin and eosin using routine procedures [25].

### 2.3. *Ex Vivo* Assay of SSH on Cultured Hepatocytes

#### 2.3.1. Cells and Reagents

HepG2, a human hepatoma cell line was grown in RPMI medium (supplemented with 10% bovine serum, 1x penicillin-streptomycin, and 1x sodium pyruvate streptomycin (HyClone Laboratories)) at 37°C in a humified chamber with 5% CO_2_.

#### 2.3.2. Hepatotoxicity and Treatment

HepG2 cells were seeded (10^5^ cells/well in triplicate) in a 96-well flat-bottom plate (Becton-Dickinson Labware) a day before treatment and grown. 2′,7′-dichlorofluorescein (DCFH) (Sigma) commonly used to measure oxidative stress, *in vitro* [26], was used as a cytotoxic agent (IC_50_: 100 *μ*M, personal observation), prepared in DMSO (Sigma). An aqueous suspension of pure SSH (250 mg/mL stock) was prepared in RPMI medium followed by diluting to seven doses of SSH (0.1, 0.25, 0.5, 1.0, 2.5, 5, and 10 mg/mL). The cells (in triplicate) were replenished with RPMI containing 100 *μ*M DCF plus a dose of SSH, including untreated as well as DCF-treated controls, and further incubated for 48 hours.

#### 2.3.3. Microscopy

A direct visual observation was made under an inverted microscope (Optica, 40x and 100x) to see any morphological changes in the cells cultured with SSH and/or DCF on day 1 and 2.

#### 2.3.4. Cell Proliferation and Viability Test

On day 2 of treatment, hepatocyte proliferation and viability test was performed using TACS MTT Cell Proliferation and Viability Assay Kit (TACS).

### 2.4. Studies of the *In Vitro* Antioxidant Activity

#### 2.4.1. DPPH Free Radical Scavenging Assay

The radical scavenging activity of the SSH against DPPH was evaluated as previously described [27]. The sample was redissolved in water, and various concentrations (31, 62, 125, 250, and 500 mg/mL) of the honey, 125 *μ*L prepared DPPH (1 mM in methanol), and 375 *μ*L solvent (methanol) were added. After 30 min incubation at 25°C, the decrease in absorbance was measured at *λ* = 517 nm. The radical scavenging activity was calculated from the equation:

(2) %  of    radical    scavenging    activity=ABScontrol−AbssampleAbscontrol×100.

#### 2.4.2. *β*-Carotene-Linoleic Acid Assay

The antioxidant activity of the SSH was evaluated, using the *β*-carotene bleaching method as described by Mothana et al. [28]. Rutin (1 mg/mL) was used as a standard. Absorbance was read at 470 nm at 15 min intervals, using a UV-visible spectrophotometer (UV mini-1240, Shimadzu, Japan). The antioxidant activity was calculated using the equation:

(3) %  of  antioxidant  activity=  1−(Abs0−Abst)  (Abs0°−Abst°)×100,

where Abs_0_ and Abs_0_° are the absorbance values measured at zero time of incubation for sample extract and control, respectively.

Abs_*t*_ and Abs_*t*_° are the absorbance values for sample extract and control, respectively, at *t* = 120 min.

### 2.5. Total Phenolic Content

The Folin-Ciocalteu method was used to determine the total phenolic content (TPC) of the honey according to Singleton et al. [29] and Liberato et al. [30]. Values of TPC were estimated by comparing the absorbance of each sample with a standard response curve generated using gallic acid (0, 12.5, 25, 50, 100, and 200 *µ*g/mL). The results were expressed as mg gallic acid equivalents (GAE)/100 g of honey. All the measurements were taken in triplicate, and the mean values were calculated.

### 2.6. Total Flavonoid Content

The total flavonoid content was determined by using a colorimetric assay as previously described [30]. Briefly, an aliquot of 5 mL of honey solution (0.02 mg/mL) or standard solution was mixed individually with the same volumes of solution of 2% aluminum chloride (AlCl_3_) and allowed to stand at room temperature for 10 minutes. The absorbance was then read at 415 nm. A calibration curve was prepared with quercetin, and the results were expressed as mg quercetin equivalents (CE)/100 g of honey.

### 2.7. Statistical Analysis

Values are given as arithmetic means ± standard error of the mean (S.E.M.). Data was statistically analyzed by using one-way analysis of variance (ANOVA) followed by Student's *t*-test.

## 3. Results

### 3.1. Acute Toxicity Test

No toxicity symptoms were recorded. The LD_50_ value by oral route could not be determined as no lethality was observed up to 5.0 g/kg of the SSH in the animals. 

### 3.2. *In Vivo* Effect of SSH on Liver and Kidney

The results indicated that animals treated with CCl_4_ showed a significant increase in all biochemical parameters tested. However, animals treated with Saudi Sidr honey for 6 weeks before the intoxication with CCl_4_ showed a significant decrease in serum GOT, GPT, ALP, GGT, creatinine, bilirubin, urea, and uric acid levels (Tables 1 and 3). CCl_4_-induced oxidative stress caused an elevation in lipid profile including cholesterol, triglycerides, LDL-C, and VLDL-C and reduction in the HDL-C levels in serum. The six-week pretreatment of rats with SSH in both doses, dose-dependently and significantly, reduced the cholesterol, triglycerides, LDL-C, and VLDL-C levels and significantly improved HDL-C level (Table 2). Silymarin, on the other hand, significantly prevent the CCl_4_-induced elevated levels of marker enzymes and lipid profile.

The results also indicated that treatment with CCl_4_ resulted in a significant increase in MDA and a significant decrease in NP-SH and TP concentration in both liver and kidney tissues (Tables 4 and 5). Treatment of rats with SSH resulted in a significantly diminished level of MDA and significantly enhanced NP-SH and TP levels in both liver and kidney tissue.

**Figure 1:**
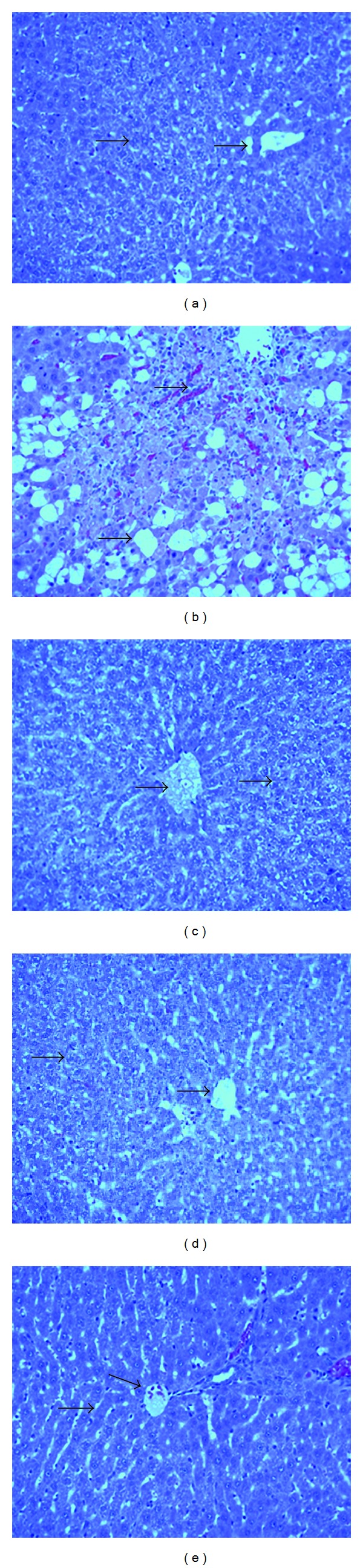
Light micrographs showing the effect of SSH on CCl_4_-induced hepatotoxicity in rats. (a) Normal hepatocytes. (b) CCl_4_-induced severe necrosis and inflammation. (c) Pretreatment of rats with SSH 0.5 g/kg. (d) Pretreatment of rats with SSH 1.0 g/kg. (e) Pretreatment of rats with silymarin 10 mg/kg.

Upon histopathological assessment of liver, the CCl_4_-induced rats showed an evidence of fatty changes with necrosis in liver cells, extensive fatty and inflammatory changes along with vascular congestion, and minimal fibrosis. The rats which received SSH (0.5 and 1.0 g/kg/day) and silymarin, as oral pretreatment showed a marked improvement in liver parenchyma with remnant degenerative changes of the cytoplasm and completely intact liver hepatocytes (Figure 1).

**Figure 2:**
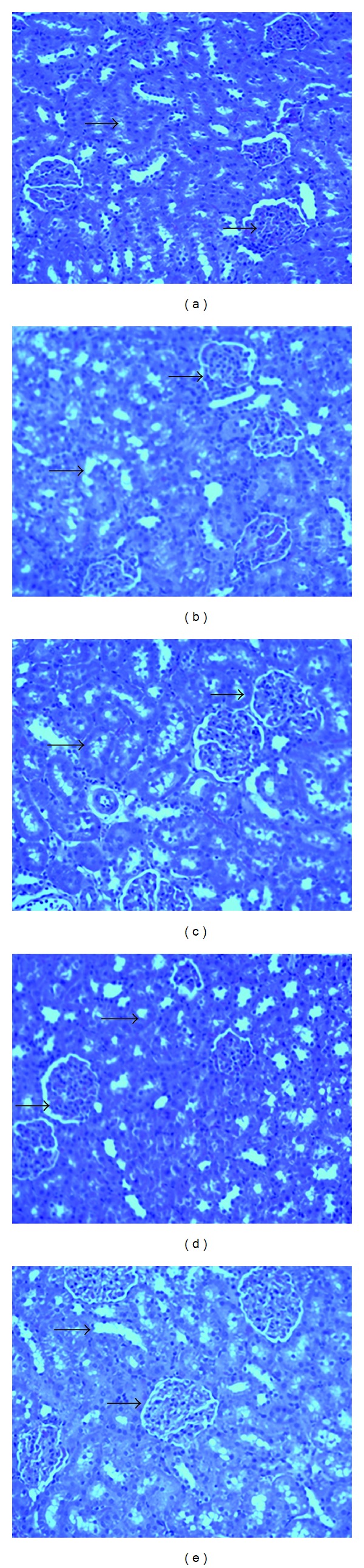
Light micrographs showing the effect of SSH on CCl_4_-induced nephrotoxicity in rats. (a) Normal kidney tissues. (b) CCl4-induced severe glomerular congestion and vacuolization of the glomerular tuft and tubules with sloughing of the renal tubular lining. (c) Pretreatment of rats with SSH 0.5 g/kg. (d) Pretreatment of rats with SSH 1.0 g/kg. (e) Pretreatment of rats with silymarin 10 mg/kg.

The histological examination of kidney sections of control rat showed normal glomeruli, interstitial tubules, and blood vessels. The kidney tissue slices in rats treated with CCl_4_ exhibited glomerular congestion with vacuolization of the glomerular tuft and tubule with sloughing of the renal tubular lining. The kidney sections in rats pretreated with honey and silymarin showed vacuolization of glomerular tuft, evidence of tubular necrosis, and, regenerative and desquamation vacuolization (Figure 2).

### 3.3. Hepatoprotective Effect of SSH on Cultured Human Liver Cells

#### 3.3.1. Microscopy 

DCF exhibited severe cytotoxic effect on the human liver cells as reflected by altered morphology compared to untreated cells. Interestingly, the DCF-treated cells supplemented with 10 mg/mL of SSH were morphologically different from the DCF-treated cells but comparable to untreated cells (data not shown).

#### 3.3.2. Hepatocyte Protection and Viability Restoration by SSH

**Figure 3:**
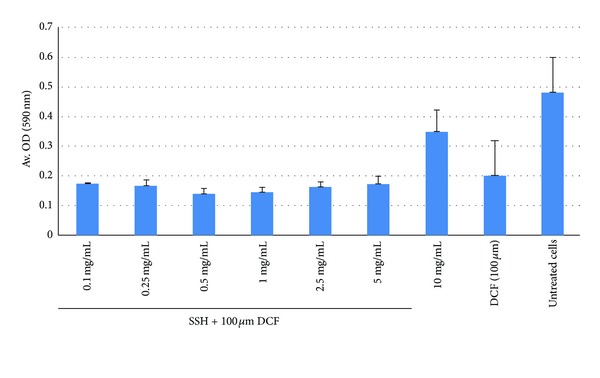
MTT test showing hepatoprotective effect of SSH (10 mg/mL) against DCF (100 *μ*M)-induced toxicity of cultured human liver cells (HepG2).

Our MTT test showed a protective effect of SSH (10 mg/mL) against DCF (100 *μ*M) induced hepatotoxicity at 48 hours posttreatment (Figure 3), and in line with our microscopic observation that confirmed the *ex vivo* hepatoprotection by pure SSH. Under these conditions DCF-toxicated cells were recovered to about 70% with 10 mg/mL of SSH. The SSH supplementation restored the hepatocytes viability by 1.5-fold against DCF toxicity.

### 3.4. Antioxidant Activity and Phenolic and Flavonoidal Contents of the SSH

The potential antioxidant activity of the SSH was investigated on the basis of DPPH radical scavenging activity and of inhibition of linoleic acid oxidation. As demonstrated in Table 6, SSH was able to reduce the stable free radical DPPH to the yellow-colored DPPH at low concentrations (125 and 250 mg/mL), almost near to the ascorbic acid. In addition to that, in the *β*-carotene/linoleic acid model system, the SSH was also able to inhibit the discoloration of *β*-carotene at a concentration of 125 mg/mL. The total antioxidant value was 90.8% (Table 6). The observed antioxidant activities were comparable to that of the positive control, rutin (Table 6). Moreover, the SSH showed high total phenolic and flavonoidal contents (105.1 ± 4.04 mg gallic acid equivalents/100 g and 48.3 ± 1.17 mg quercetin equivalents/100 g).

## 4. Discussion

The liver is an organ that not only performs physiological functions but also protect against the hazards of harmful drugs, chemicalss and xenobiotics. The liver is one of the largest and highly complex organs with multifunction, including nutrient storage, maintenance of homeostasis, secretory and excretory function, and synthesis of proteins [31]. These functions also include the metabolism of certain hormones, lipid metabolism (cholesterol, triglycerides, and high-density lipoproteins (HDL)), protein metabolism, and detoxification of xenobiotics [32]. The model used in the present study has been subjected to thorough critical appraisal and validated animal models of hepatorenal protective activity of honey in rats against CCl_4_ as a hepatorenal toxin to prove its claims in folklore practice against liver and kidney disorders.

Carbon tetrachloride, besides exerting its toxic effect on liver, also reportedly gets distributed at higher concentrations in the kidney than in the liver [33]. The mechanism of CCl_4_ renal toxicity is almost the same as that of liver, but CCl_4_ shows a high affinity to the kidney cortex which contains cytochrome P-450 predominantly [34, 35]. Due to CCl_4_ hepatorenal injury, the transport function of hepatocytes and nephrotic cells gets disturbed resulting in the leakage of plasma membrane, thereby causing an increased enzyme level in the serum [36].

In the present study CCl_4_ administration to rats caused a significant increase in serum GOT, GPT, GGT, ALP, urea, uric acid, bilirubin, and creatinine as well as MDA in the liver and kidney levels accompanied with a significant decrease in total protein and NP-SH in liver and kidney, which indicates the extensive disruption of the structure and function of the liver and kidney. The tendency of these marker enzymes (SGPT, SGOT, ALPG, GGT) at a near normal level in the groups of rats treated with Saudi Sidr honey and silymarin is a clear manifestation of antihepatorenal toxic effect of the honey. Our findings are in agreement with an earlier study in which feeding of honey caused liver protective effect by depleting the elevated liver marker enzymes in CCl_4_-treated rats [37]. On the other hand, treatment of rats with CCl_4_ increases the levels of total lipids, triglycerides, and cholesterol in serum [38]. The significant diminution of lipids and total cholesterol in the SSH-treated rats and an increase in HDL-C level further indicates the hepatoprotective potential of the honey.

Furthermore, the efficacy of any hepatoprotective drug is essentially dependent on its capacity of either maintaining normal physiological function or diminishing the harmful effects which were affected by hepatotoxic agents [39]. Reduction in total protein (TP) content can be deemed as a useful index of severity of hepatocellular damage [40]. The lowered levels of total proteins in the serum of CCl_4_-treated rats exhibited the severity of hepatopathy. Thus, this suggests that honey possesses the ability to promote protein synthesis leading to the higher concentration of protein in the liver [35].

The present study also revealed that the administration of CCl_4_ caused marked impairment in renal function alongside with significant oxidative stress in the kidney [41]. Serum creatinine, urea, and uric acid concentrations were significantly higher in CCl_4_-treated rats. Serum creatinine elevation was caused by CCl_4_ due to altered kidney function. Urea is the main end product of protein catabolism. It is one of the waste products of the body which is passed into blood stream to be removed by kidney [42]. Honey significantly decreased the elevated levels of serum creatinine, urea, and uric acid, which indicates that the honey possibly protects kidney tissue against oxidative damages induced by CCl_4_ [43] and indicates maintenance of renal function [44].

*In vitro* antioxidant activity of Saudi Sidr honey revealed a strong antioxidant activity in both the tests DPPH and *β*-carotene linoleic acid used. The observed hepatonephro protective and antioxidant activity of honey might have been due to the presence of phenolic and flavonoidal contents. In the current study, the increase in MDA level in the liver and kidney by CCl_4_ suggests enhanced lipid peroxidation (LPO) which is an important pathogenic event that damages biomembranes [45]. LPO is thought to be a consequence of oxidative stress which occurs when the dynamic balance between prooxidant and antioxidant mechanism is impaired [46]. Malondialdehyde is known to be a reliable marker of LPO and oxidative stress [47]. Treatment of rats with SSH significantly prevented CCl_4_-induced increase in MDA concentration in both liver and kidney. Hence, the observed hepato-renal protective activity of honey may be due to its antioxidant property.

Nonprotein sulfhydryls are known to be involved in several defense processes against oxidative damage; protects cells against free radicals peroxides and various poisonous substances [48]. Thus, a deficiency of GSH within the living organisms can cause tissue injury and malfunction [49]. In the current study, the liver and kidney NP-SH level in CCl_4_-treated group was significantly diminished when compared with the control group. These findings are in accordance with earlier reports as sulfhydryl levels were significantly depleted in different organs of rats, when exposed with CCl_4_ [50]. Pretreatment of rats with SSH replenished NP-SH concentration in both liver and kidney homogenate as compared with CCl_4_ only treated animals, suggesting the ameliorative effects of SSH on liver and kidney damage induced by CCl_4_, at least in part, due to its free radical scavenging activity. Furthermore, most of the parameters in the group which received CCl_4_ plus Sidr honey having nearer value of the control group demonstrate Sidr honey to have antiradical effect [51]. This effect is considered to be related to the phenolic compounds and *in vitro* antioxidant activity of Saudi Sidr honey.

On the other hand, DCFH is generally used to measure *in vitro* oxidative stress generated by free radicals through the principle of oxidation of DCFH to the fluorescent DCF. However, we used this agent because of its cytotoxic effect (unpublished). In this study, our *ex vivo* SSH protection against DCFH-induced human liver toxicity further supported the *in vivo* effects of SSH in CCl_4_-induced rat liver injury. In this way, we have confirmed our results by using two different toxins in two different systems, at least for hepatoprotection.

## 5. Conclusions

In conclusion, the administration of Saudi Sidr honey (SSH) prevented biochemical and histomorphological alteration induced by CCl_4_. This hepatorenal protective effect of SSH could be attributed to the presence of antioxidative factors for example, phenolic and flavonoidal compounds which cause significant lowering of the oxidative threat leading to normal physiological function. The findings support the use of SSH for the treatment of several liver and kidney ailments.

## Figures and Tables

**Table 1:** Effect of honey on CCl_4_-induced hepatotoxicity-related parameters in rats.

Treatment group (*n* = 6)	SGOT (U/L)	SGPT (U/L)	GGT (U/L)	ALP (U/L)	Bilirubin (mg/dL)
Normal control	75.53 ± 2.86	26.93 ± 1.75	3.66 ± 0.37	282.00 ± 10.05	0.52 ± 0.02
CCl_4 _only (1.25 mL/kg)	300.00 ± 8.23^∗∗∗a^	209.50 ± 8.44^∗a^	15.45 ± 1.20^∗∗∗a^	671.33 ± 15.15^∗∗∗a^	3.45 ± 0.16^∗∗∗a^
Honey (0.5 g/kg) + CCl_4_	273.66 ± 10.28^b^	182.83 ± 7.61^∗b^	12.81 ± 0.61^b^	614.83 ± 13.41^∗b^	2.67 ± 0.14^∗∗b^
Honey (1.0 g/kg) + CCl_4_	200.00 ± 5.08^∗∗∗b^	140.83 ± 6.12^∗∗∗b^	10.38 ± 0.54^∗∗b^	605.16 ± 12.82^∗∗b^	2.01 ± 0.10^∗∗∗b^
Silymarin (10 mg/kg) + CCl_4_	133.58 ± 8.08^∗∗∗b^	106.78 ± 6.33^∗∗∗b^	5.08 ± 0.30^∗∗∗b^	416.16 ± 11.62^∗∗∗b^	1.17 ± 0.10^∗∗∗b^

All values represent mean ± SEM. *P < 0.05; **P < 0.01; ***P < 0.001; ANOVA, followed by Dunnett's multiple comparison test.

^a^As compared with normal group. ^b^As compared with CCl_4 _only group.

**Table 2:** Effect of honey on CCl_4_-induced lipid profile changes in rats.

Treatment group (*n* = 6)	Cholesterol (mg/dL)	Triglycerides (mg/dL)	HDL (mg/dL)	LDL (mg/dL)	VLDL (mg/dL)
Normal control	13.79 ± 1.22	91.34 ± 4.57	52.56 ± 6.14	68.98 ± 6.14	105.33 ± 3.81
CCl_4 _only (1.25 mL/kg)	43.61 ± 1.58^∗∗∗a^	237.63 ± 10.61^∗∗∗a^	23.47 ± 1.09^∗∗∗a^	218.05 ± 7.94^∗∗∗a^	281.33 ± 11.01^∗∗∗a^
Honey (0.5 g/kg) + CCl_4_	34.35 ± 1.63^∗∗b^	172.29 ± 8.93^∗∗∗b^	31.28 ± 1.12^∗∗b^	171.29 ± 8.19^∗∗b^	206.66 ± 9.77^∗∗∗b^
Honey (1.0 g/kg) + CCl_4_	20.00 ± 1.17^∗∗∗b^	170.50 ± 6.46^∗∗∗b^	44.38 ± 1.97^∗∗∗b^	100.00 ± 5.87^∗∗∗b^	190.66 ± 6.97^∗∗∗b^
Silymarin (10 mg/kg) + CCl_4_	38.79 ± 0.80^∗b^	198.43 ± 9.82^∗b^	29.27 ± 1.55^∗b^	199.98 ± 4.02^∗b^	237.33 ± 9.16^∗b^

All values represent mean ± SEM. *P < 0.05; **P < 0.01; ***P < 0.001; ANOVA, followed by Dunnett's multiple comparison test.

^a^As compared with normal group, ^b^As compared with CCl_4 _only group.

**Table 3:** Effect of honey on CCl_4_-induced kidney function test in serum.

Treatment group (*n* = 6)	Creatinine (mg/dL)	Uric Acid (mg/dL)	Urea (mmol/L)	Sodium (mEq/L)	Potassium (mEq/L)	Calcium (mg/dL)
Normal control	1.34 ± 0.09	1.28 ± 0.010	35.8 ± 1.90	54.96 ± 0.99	3.61 ± 0.18	4.42 ± 0.34
CCl_4 _only (1.25 mL/kg)	7.48 ± 0.31^∗∗∗a^	5.68 ± 0.36^∗∗∗a^	167.16 ± 4.65^∗∗∗a^	101.89 ± 2.26^∗∗∗a^	10.58 ± 0.48^∗∗∗a^	24.64 ± 0.81^∗∗∗a^
Honey (0.5 g/kg) + CCl_4_	6.48 ± 0.31^∗b^	4.25 ± 0.43^∗b^	157.5 ± 5.51^b^	88.67 ± 2.35^∗∗b^	9.18 ± 0.27^∗b^	20.42 ± 0.98^∗∗b^
Honey (1.0 g/kg) + CCl_4_	5.11 ± 0.13^∗∗∗b^	2.89 ± 0.21^∗∗∗b^	131.83 ± 8.15^∗∗b^	78.14 ± 2.30^∗∗∗b^	7.31 ± 0.23^∗∗∗b^	13.76 ± 0.87***
Silymarin (10 mg/kg) + CCl_4_	6.08 ± 0.27^∗∗b^	4.30 ± 0.25^∗b^	154.66 ± 5.85^b^	93.44 ± 3.20^∗b^	10.05 ± 0.40^b^	25.12 ± 0.84^b^

All values represent mean ± SEM. *P < 0.05; **P < 0.01; ***P < 0.001; ANOVA, followed by Dunnett's multiple comparison test.

^a^As compared with normal group, ^b^As compared with CCl_4 _only group.

**Table 4:** Biochemical parameters (liver tissue) treated with honey.

Treatment group (*n* = 6)	Total protein (g/L)	MDA (nmol/g)	NP-SH (nmol/g)
Normal control	122.49 ± 4.61	0.97 ± 0.11	7.08 ± 0.34
CCl_4 _only (1.25 mL/kg)	41.52 ± 2.59^∗∗∗a^	11.88 ± 0.77^∗∗∗a^	3.81 ± 0.43^∗∗∗a^
Honey (0.5 g/kg) + CCl_4_	54.38 ± 4.29^∗b^	7.96 ± 0.77^∗∗b^	4.96 ± 0.47^b^
Honey (1.0 g/kg) + CCl_4_	58.44 ± 3.81^∗∗b^	5.80 ± 0.58^∗∗∗b^	6.13 ± 0.37^∗∗b^
Silymarin (10 mg/kg) + CCl_4_	80.83 ± 3.28^∗∗∗b^	2.91 ± 0.23^∗∗∗b^	6.96 ± 0.29^∗∗∗b^

All values represent mean ± SEM. *P < 0.05; **P < 0.01; ***P < 0.001; ANOVA, followed by Dunnett's multiple comparison test.

^a^As compared with normal group, ^b^As compared with CCl_4 _only group.

**Table 5:** Biochemical parameters (kidney tissue) treated with honey.

Treatment group (*n* = 6)	Total protein (g/L)	MDA (nmol/g)	NP-SH (nmol/g)
Normal control	89.75 ± 4.12	0.69 ± 0.13	7.23 ± 0.46
CCl_4 _only (1.25 mL/kg)	31.70 ± 3.45^∗∗∗a^	8.53 ± 0.91^∗∗∗a^	4.08 ± 0.35^∗∗∗a^
Honey (0.5 g/kg) + CCl_4_	41.89 ± 1.62^∗b^	5.77 ± 0.39^∗b^	4.76 ± 0.45^b^
Honey (1.0 g/kg) + CCl_4_	46.17 ± 2.18^∗∗b^	3.95 ± 0.32^∗∗∗b^	5.80 ± 0.31^∗∗b^
Silymarin (10 mg/kg) + CCl_4_	64.72 ± 7.42^∗∗∗b^	1.89 ± 0.28^∗∗∗b^	6.29 ± 0.46^∗∗b^

All values represent mean ± SEM. *P < 0.05; **P < 0.01; ***P < 0.001; ANOVA, followed by Dunnett's multiple comparison test.

^a^As compared with normal group, ^b^As compared with CCl_4 _only group.

**Table 6:** Free radical scavenging activity, antioxidant activity, total phenolic, and total flavonoidal contents of the honey sample.

Plant species	Radical scavenging activity (%)					Total antioxidant activity (%)	TPC (mg GAE /100 g)	TFC (mg QE/100 g)
	31.25	62.5	125	250	500	125 (mg/mL)
Honey	28.0	45.0	72.7	89.3	91.8	90.5 ± 7.62	105.1 ± 4.04	48.3 ± 1.17
Ascorbic acid	18.5	74.1	88.9	93.0	94.0	—		
Rutin						91.9 ± 5.83		

TPC: Total phenolic content; TFC: Total flavonoidal content.
